# Infant Feeding

**Published:** 1902-08-09

**Authors:** 


### INFANT FEEDING.

In opening a discussion on the modification of milk in the treatment of infants, Professor Rotch, of Harvard University, urged that the exact analytical methods of modern chemistry now rendered it possible for the physician to free himself from the empirical methods of the nurse, and from the tyranny of the manufacturer of artificial foods, and to prescribe the nutrition of his infant patients with the same exactness and knowledge which he adopted in the administration of drugs. Although there could be no doubt that the best form of food for young infants was the natural supply of breast milk, circumstances at times compelled the physician to find a substitute for this, and in so doing it was necessary to remember that human milk differed widely in its quantitative composition. The physician must therefore be able, if he was to copy nature's methods, to vary the percentage of the proximate principles in the substitute food according to the requirements of the individual. The difficulty in providing a proper food for infants arose, then, from the fact that every specimen of milk used for the purpose required modifying. This modification was usually effected by a rough and empirical process, but it was very desirable to replace this by a precise and definite proceeding, so that the physician should be able to know the percentage composition of the food taken by the child, and should be able to vary this as might be necessary. The first essential was pure milk of good quality, of known quantitative value, and free from bacterial contamination; the second was the safe transmission of such milk from its source to the infant; and the third was the capacity to vary the composition of the food as the judgment of the physician directed, and for the latter purpose he strongly advocated the establishment of milk laboratories, where the processes of milk modification could be conducted with the same intelligence and care as ruled the conduct of the pharmacy. By certain modifications of method the same principles might be adopted in the home, but Professor Rotch held that the necessary modification could only be properly carried out on a quantitative basis, and he urged that these ends could be best attained by some such methods as he had described.

The discussion which followed this paper was interesting, the conclusions arrived at by Professor Rotch being by no means universally accepted. On all hands he was supported as to the necessity for milk being pure and uncontaminated, but the necessity for the exact modification, and especially the "laboratory" modification, was not so generally admitted. Dr. De Rothschild, of Paris, said that healthy children were readily nourished on pure cow's milk, and this by appropriate dilution could be adapted to most cases of disease. The essential thing was to be able to purchase pure milk, and if this could be done all the necessary preparation could be conducted at home under the physician's direction. Dr. Northup, of New York, supported Professor Rotch, holding that it was necessary to secure food adapted to the individual infant's requirements, and the opportunity of obtaining this in an exact fashion he regarded as a triumph of American medicine. Dr. George Carpenter held that the question of expense had to be considered, and this he feared was a practical difficulty. Dr. Eric Pritchard urged that the less interference that milk used for infant feeding was subjected to the better. The fact that mother's milk varied from time to time was, he thought, an argument against enforcing too rigid a standard in preparing a substitute. The expense of laboratory milk was a serious objection, and he held that if cream of a known strength could be obtained all necessary modifications in the milk required could be carried out at home without the aid of a laboratory. Dr. Sandwith, of Cairo, and Professor Byers, of Belfast, supported Professor Rotch.

Professor Rotch, in reply, said that his proposal was not to substitute artificial for natural feeding, but to make the former, when it was required, an exact instead of a chance proceeding. The question of expense, he thought, would not be allowed to block a reform which concerned the well-being of infant life and growth.

